# An investigation of perceived vehicle speed from a driver's perspective

**DOI:** 10.1371/journal.pone.0185347

**Published:** 2017-10-17

**Authors:** Changxu Wu, Dekuang Yu, Amy Doherty, Tianyi Zhang, Leo Kust, Gang Luo

**Affiliations:** 1 Center for Psychological Science at Zhejiang University, Hangzhou, Zhejiang Province, China; 2 Southern Medical University, Guangzhou, Guangdong Province, China; 3 Harvard Medical School, Boston, Massachusetts, United States of America; 4 University of Arizona, Tucson, Arizona, United States of America; 5 University at Buffalo—The State University of New York, Buffalo, New York, United States of America; Beihang University, CHINA

## Abstract

**Purpose:**

Speed estimation of drivers’ own vehicles and other vehicles on the road is an important task for drivers and is also crucial to the roadway safety. The objective of the study was to examine the effects of multiple factors such as image scale, speed, road type, driving experience, and gender on the speed perception of drivers’ own vehicles.

**Methods:**

Thirty participants consisted of 17 males and 13 females, including 13 without driving experience. All participants estimated the driving speed of 192 5-second video clips, which were selected from naturalistic driving recordings. The recorded driving speeds were evenly distributed across the entire range from 5mph to 65mph. Half of the selected video clips were recorded on wide roads and another half were recorded on comparatively narrow roads. Video clips were played on a large screen, with each clip shown in one of 4 image scales (100%, 75%, 50%, and 38% of the actual field of view in the real world).

**Results:**

Speed estimates were most accurate for the smallest image size (38% of the actual field of view). As the image size increased, the driving speed was increasingly underestimated. Participants with driving experience accurately estimated the driving speed on both wide and narrow roads whereas those without driving experience had greater underestimates on wider roads. Speeds were most accurately estimated within the range 25-35mph, but the speeds slower than the range tend to be overestimated and the speeds faster than the range are more likely to be underestimated. While males and females showed the same pattern across speed groups, females have greater estimation errors at the highest and lowest speed groups. Participants without driving experience showed increasing underestimation of speed as driving speed increased whereas participants with driving experience primarily underestimated the highest speeds.

**Conclusions:**

The present study shows the effect of multidimensional influential factors on perceived vehicle speed from drivers’ perspective. The results also have implications for driving simulation scenario design, driving simulator setup, and the assessment of speed control in simulated and naturalistic environments.

## Introduction

Perceiving the speed of drivers’ own vehicles and other vehicles is critical to ensure safe driving and, in particular, that the maneuvering actions do not violate the law, such as not going over the speed limit unintentionally and promptly maneuvering vehicles to avoid accidents. Speedometers serving as a direct and reliable information resource usually provide drivers with accurate speed indication of their own vehicles; however, in some cases, drivers may not be able to refer to the speedometer when the complex traffic situation imposes high cognitive workload on them. Speed estimation, therefore, becomes an important component of safe driving beyond monitoring the road traffic.

In the existing literature concerning about the speed estimation, the majority of work has focused on the perception of drivers’ own vehicle’s speed [1–8]. For example, Ben-Bassat and Shinar (2011) [1] tested the combined effects of three roadway design elements—roadway shoulder width, guardrail existence, and roadway geometry (curvature)—on perceived safe driving speed and estimated road safety. They found that roadway geometry can be used to reduce driving speeds, but at the same time it can have a negative effect on maintaining a stable lane position in sharp curves. Moreover, Fildes & Lee (1993) [3] found that people usually drive at higher speed on the road they perceive to be wide. Fildes et al., (1989) [4] discussed that the interaction of driving experience, road type and drivers’ gender has effect on speed estimation.

Relatively few studies have been carried out to quantify subjective estimation of vehicle speed from the pedestrian perspective. In the study conducted by Troscianko et al. (1999) [9], ten participants were asked to estimate the speed of a vehicle in real traffic from an interior site that generally eliminated nonvisual cues. The results showed that participants, from the pedestrians’ perspective, significantly underestimated vehicle’s speed. Sun et al., (2015) [10] recently conducted another field study in a naturalistic traffic environment and found an effect of actual speed and weather condition on the subjective estimation of vehicle speed. In sunny conditions, pedestrians tended to underestimate actual vehicle speeds that were higher than 40 km/h but were able to accurately estimate speeds that were lower than 40 km/h. In rainy conditions, pedestrians tended to underestimate actual vehicle speeds that were higher than 45 km/h but were able to accurately estimate speeds ranging from 35 km/h to 45 km/h [10]. The implications from the experiments have been drawn for our experiment design such that the actual driving speed is considered as a potential factor that could influence the perceived speed. We formulated the hypothesis that speed estimation accuracy may depend on speed range; for example, drivers may overestimate the speed at lower range of driving speeds and underestimate the speed at higher range of driving speeds. The results might also help interpret the contradictory findings among previous driving simulator validation studies [11–12]. Godley et tal., (2002) [11] found that, on two-lane roads with 37.5 mph (60 km/h) speed limit, participants generally drove slower in the simulator than in the instrumented car, but Tornros (1998) [12] found that participants drove faster (i.e. usually higher than 50 mph (80 km/h)) in simulated tunnels than in real tunnels and elimination of speed information from the speedometer caused a small speed increase in both simulated and real situations. The different driving tendency between the real life and the simulated situations found in two articles might be explained by the speed perception error at different speeds, which could potentially impact the absolute validity of a driving simulator.

The perception of speed of movement is a basic visual function. Vision science studies show that optic flow is one of the main visual cues used for speed perception [13–14] and can be used to reliably estimate travel distance [15]. Although Wood & Troutbeck (1992) [16] found the constriction of the binocular visual field did not significantly affect speed estimation of drivers’ own vehicle, a few studies have been conducted to point out the effect the field of view on the perception of other objects’ speed. Osaka (1988) [17] found that the perceived speed was underestimated as field of view was reduced. The result is also evidenced by the experiment conducted by Pretto, Ogier, Bülthoff and Bresciani (2009) [18]. In their study, Pretto et al., (2009) [18] had subjects view two intervals of constant, forward visual movement through a volume of random dots, which formed a constant visual flow resembling self-motion, and the subjects were then asked to indicate the interval with faster speed. The results showed that subjects accurately estimated the speed of dot movement when the field of view was not restricted. However, when peripheral vision was occluded, visual speed was underestimated with underestimation increasing as the size of the field of view decreased from 60° to 10°. When central vision was occluded, visual speed was overestimated, i.e. the comparison stimulus was perceived faster than the standard. In this case, there was no difference across the size of the central field occluded. The sensitivity in terms of the precision in measurement did not differ across the different field of views. Pretto et al., (2009) [18] speculated that “during forward self-motion with a restricted Field of View (FOV), the availability of low angular velocities in the central area of the FOV directly decreases the estimated speed”. In our study, we examined the effect of field of view on speed perception by varying the displayed image sizes. The major difference between our experiment and theirs is that we used naturalistic driving recordings rather than the visual flow formed by random dots. Same as in their study, the angular velocities in the central are of FOV was lower when image size was smaller.

Based on the literature review above, previous studies were mainly limited to the investigation of the interactions among different factors that have effect on speed perception of drivers’ own vehicles. There is a need to explore more factors, such as the road type, gender, driving experience, speed, and displayed image scale, that could affect speed perception of other vehicles from drivers’ perspective in the context of real-road driving. In real world situations, basic visual functions are not the only factors, and high-level cognition always plays an important role. For instance, drivers may utilize their experiences for making their judgments Therefore, videos recorded in natural driving activities were used in this study to allow participants to access visual information very similar to what they would receive in driving, minimizing the compromise in the validity of the visual stimuli.

## Methods

### Participants

Thirty participants (17 males and 13 females, mean age 36.0 years, age standard deviation16.9 years) volunteered in the study. All participants had normal or corrected-to-normal vision (20/40 or better). Thirteen participants had no driving experience. The other 17 participants had an average of 17.9 years driving experience ranging from 1 to 65 years. The study was approved by the institutional review board at Schepens Eye Research Institute and conducted in accordance with the tenets of the Declaration of Helsinki. All participants gave voluntary written informed consent after explanation of the study.

### Task and experimental procedures

**Fig 1:**
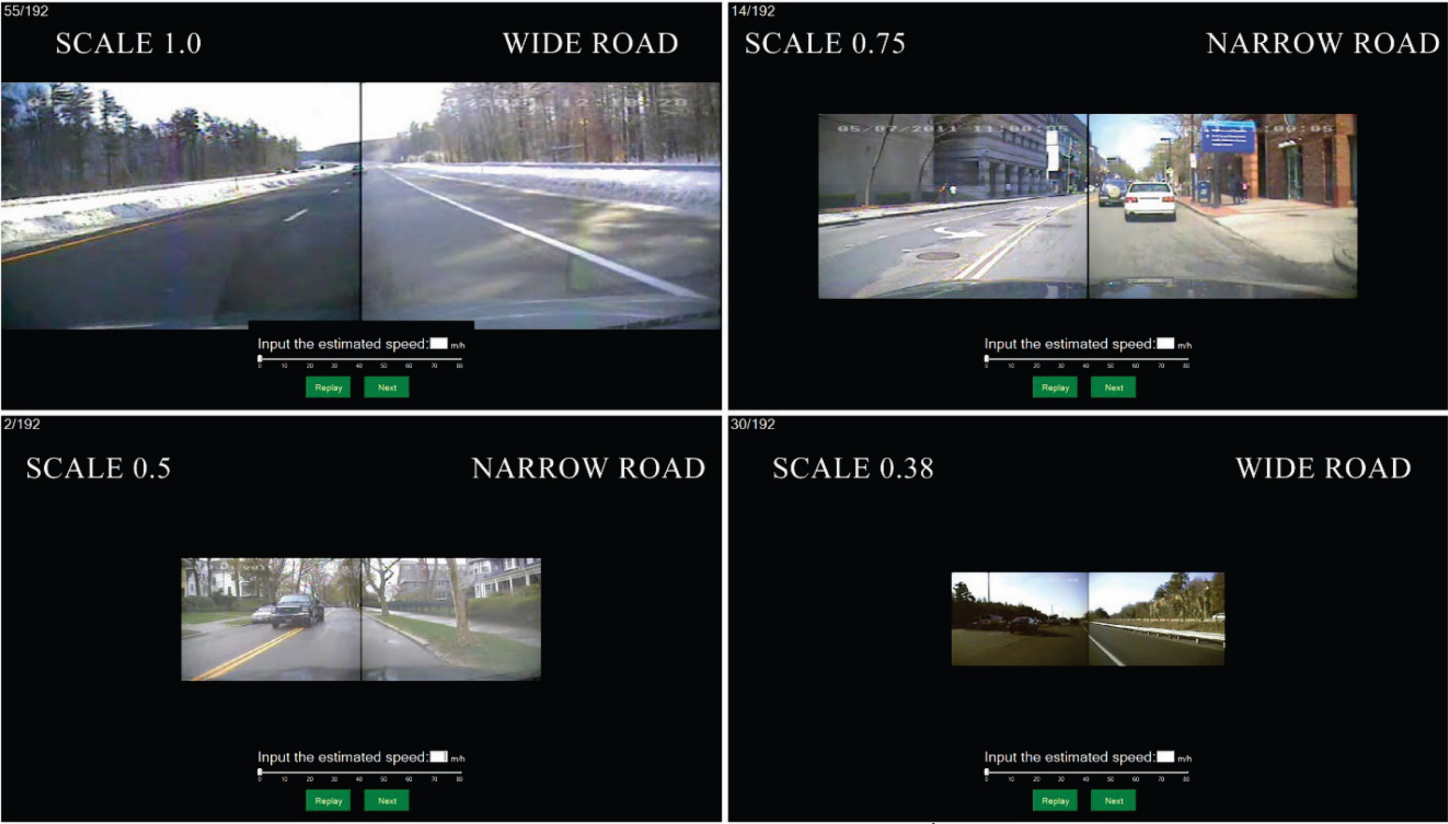
Interface for driving speed estimation system showing examples of the narrow and wide road types and 4 image scales (upper left-scale 1.0, upper right-scale 0.75, lower left-scale 0.5, lower right-scale 0.38).

To study the effect of video image size on speed judgment, participants were asked to estimate the speed (in terms of miles per hour) of 192 video clips presented in random order on a 56-inch Digital Light Projection (DLP) HDTV (Samsung HLT5687S) with a resolution of 1920×1080 pixels. Participants sat in a dark room 25 inches from the screen with their eye height level with the center of the screen. Five sample driving videos were presented with the driving speed value displayed on the screen, as training runs prior to the 192 test video clips. All 4 sizes were presented to the subjects in the training. After each 5-second clip was presented, a speed estimation pane appeared and participants estimated the speed of the video by entering a value by keyboard or dragging the bar shown on the screen (Fig 1). During the test session, participants could pause and take a 2-min break whenever they felt tired.

### Visual stimuli

Stimuli were 192 video clips taken from a naturalistic driving video inventory collected using an in-car camera system [19]. They were displayed on the TV at 4 different sizes: width of the video in full screen (1.0), 75% of the screen (0.75), 50% of the screen (0.5), and 38% of the screen (0.38). At full screen size, the horizontal field of view of the TV screen spanned 88 degrees for viewers sitting at 25-inch distance. The screen FOV was the same as the video contents in the real world, which were captured by two side-by-side cameras [19].

Video clips were classified into two categories (wide and narrow roads, see Fig 1) according to the criteria that wide roads include 3 or more lanes with open surroundings with buildings, trees and hills etc. at far distances, and narrow roads include less than 3 lanes with buildings, trees, and other vehicles at close distances. Each driving video clip lasted 5 seconds with near constant driving speed (the range of speed fluctuation was less than 1 mph). The driving speeds in the 192 video clips ranged from 5 miles/hour to 65 miles/hour and were divided into 6 speed groups in 10 mile/hour increments. Video clips in each speed group were carefully balanced across image size and road category (Table 1) to maximize stimulus homogeneity. ANOVA confirmed that the stimulus speed was not significantly different across image size (F_3,144_ = 0.972, p = 0.408) or road category (F_1,144_<0.001, p = 0.995). The offset of stimulus speed from speed group center (e.g. the offset of a 8mph stimulus from its group center, 10mph, is -2mph) was not different across group (F_5,186_<0.131, p = 0.985).

**Table 1:** The distribution of the 192 driving video clips. Numbers in parentheses are speed mean and standard deviation of the column or row.

Road Type	Image size	Driving speed (unit: miles/hour)						Total
		5–15	15–25	25–35	35–45	45–55	55–65	
Wide road	Scale 1	4	4	4	4	4	4	24
								(34.4±17.7)
	Scale 0.75	4	4	4	4	4	4	24
								(35.5±17.8)
	Scale 0.5	4	4	4	4	4	4	24
								(35.0±17.8)
	Scale 0.38	4	4	4	4	4	4	24
								(34.9±17.9)
Narrow road	Scale 1	4	4	4	4	4	4	24
								(34.3±17.6)
	Scale 0.75	4	4	4	4	4	4	24
								(35.5±17.7)
	Scale 0.5	4	4	4	4	4	4	24
								(34.9±17.7)
	Scale 0.38	4	4	4	4	4	4	24
								(35.0±17.5)
Total		32	32	32	32	32	32	192
		(9.8±3.0)	(19.7±2.7)	(30.1±3.0)	(40.2±2.9)	(49.9±2.7)	(59.8±2.7)	

### Driving video inventory

Video clips were selected from a driving video inventory composed of 765 five-second driving video clips. The inventory was built from naturalistic driving videos recorded in the Boston, MA area using a digital video recorder (DVR) [19–20]. Vehicle speed was recorded by the DVR. To extract the 5-second driving videos from the footage, a driving speed analysis program and driving video generation software were developed. The driving speed analysis program selected 5-second long segments of constant speed, and exported the video segment information. The driving video generation program extracted the driving video clips according to the video segment information.

### Data analysis

Estimation error was computed by subtracting the real vehicle speed from participants’ estimated speed with negative values representing an underestimation of speed and positive values an overestimation. Using SPSS v24, we conducted repeated measures ANOVA to test the effects of image size, road type, driving speed, gender, driving experience, and interactions between these factors. For descriptive statistics, values in the text and all figures are the mean ± 95% confidence interval, unless otherwise noted.

## Results

In summary, the effects of image size, road type, driving speed, and driving experience were statistically significant. The gender effect approached statistical significance (p = 0.058). Also, the interactions of driving experience with road type, driving speed with gender, and driving speed with driving experience were significant as well. These effects are presented in detail below.

### Video image size

**Fig 2:**
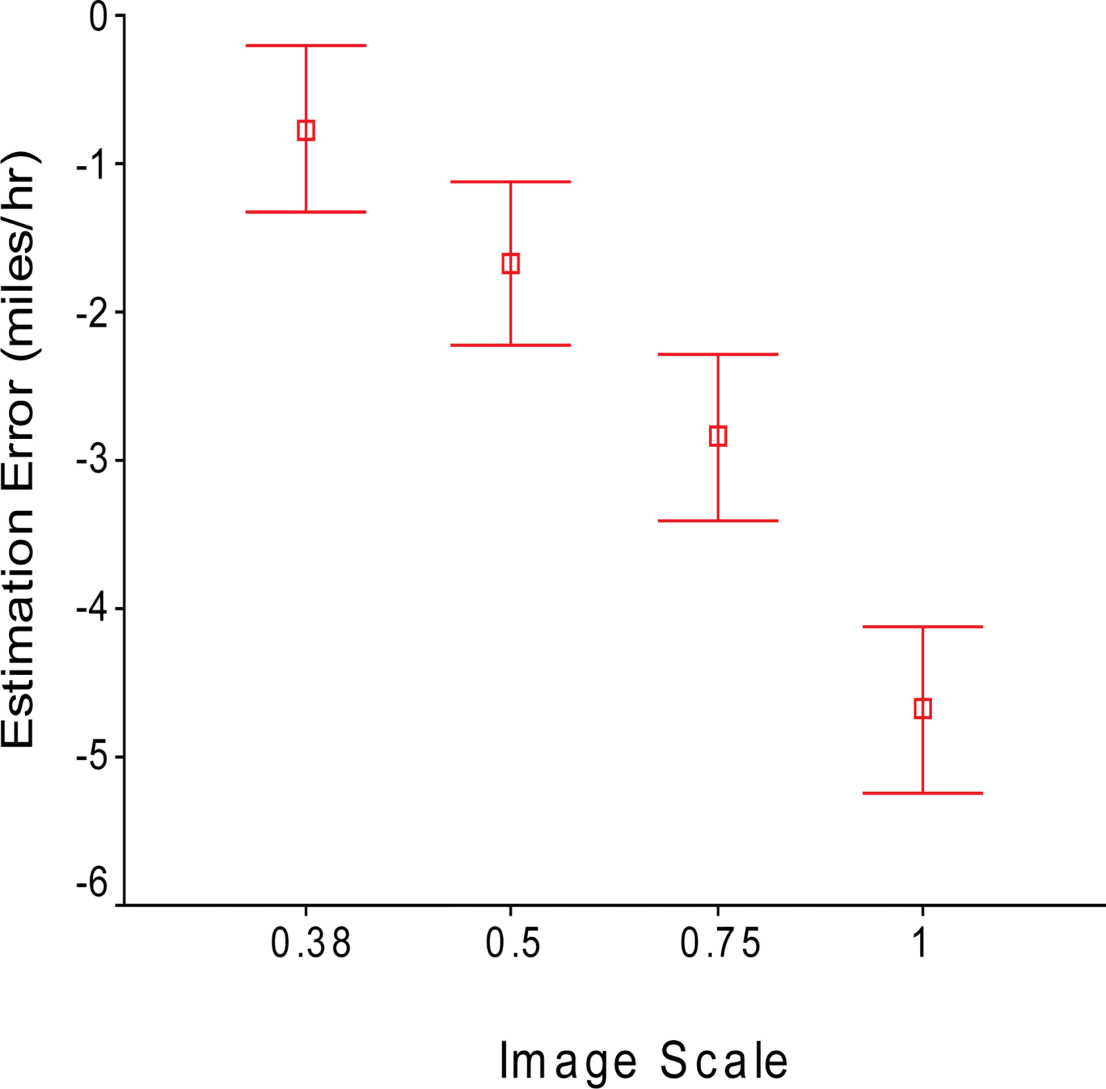
Underestimation of speed increases with increasing image size.

There was a significant effect of image size on estimation error (Fig 2; F_3, 78_ = 45.5, p<0.001). Speeds were most accurately estimated for scale 0.38—the average error was only -0.81 miles/hour. Underestimation increased with increasing image size. The average error of speed estimation with image scale 1 was -4.6 miles/hour.

### Road type and driving experience

**Fig 3:**
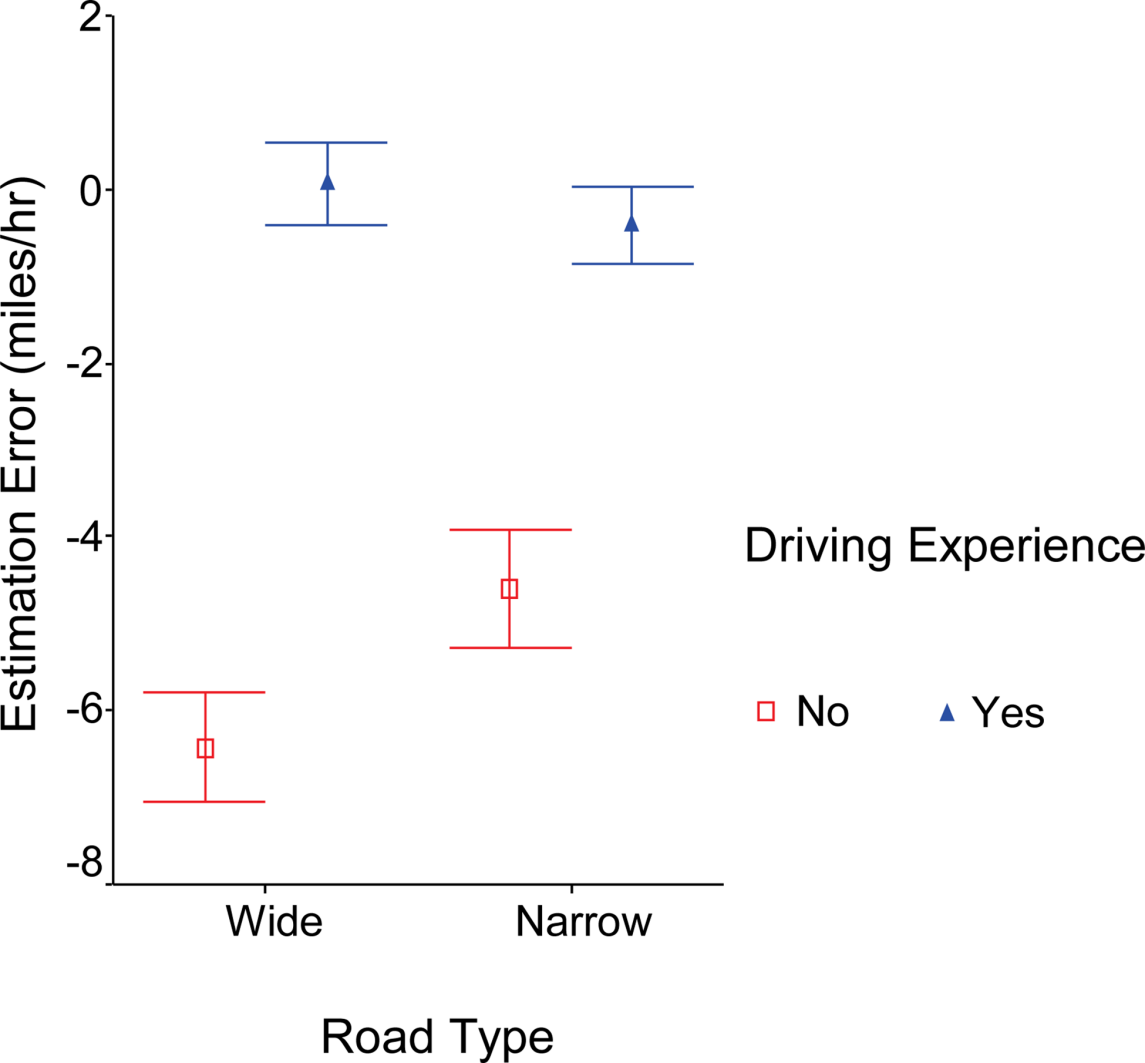
Participants with driving experience accurately estimated speed for wide and narrow roads whereas participants without driving experience underestimated speed with greater underestimation on wide roads.

Overall, participants with no driving experience perceived vehicle speed significantly slower than participants with driving experience (Fig 3; -6.16 vs 1.24, F_1,26_ = 11.2, p = 0.003). While road type did not affect the estimation of participants with driving experience (F_1,15_ = 0.27, p = 0.61), participants with no driving experience underestimated speed with significantly larger error for wide roads than narrow roads (-6.4 vs -4.6, F_1,11_ = 6.3, p = 0.03).

### Driving speed

**Fig 4:**
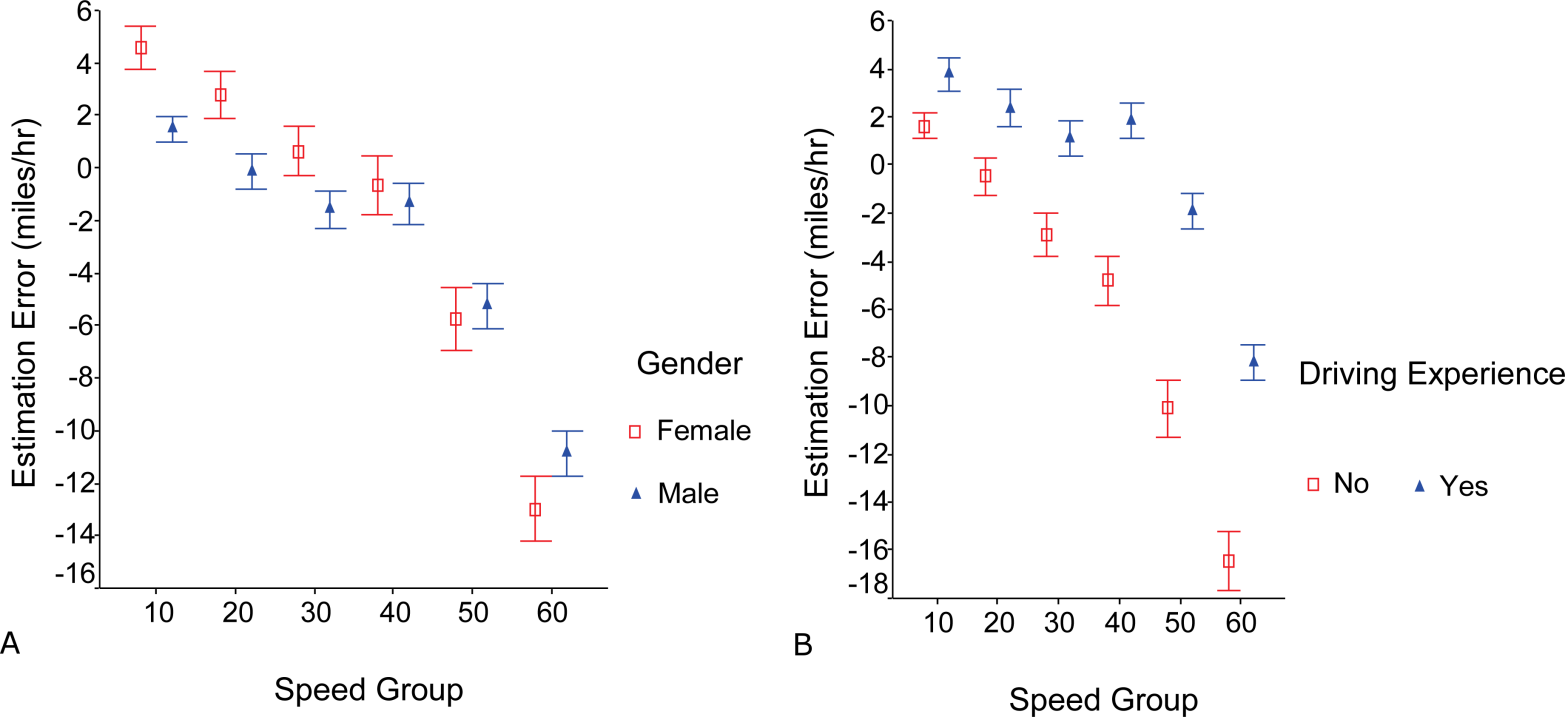
Effects of gender and driving experience on speed estimation. (a) Females had greater estimation errors than males with the greatest differences at the highest and lowest speed groups. (b) Participants without driving experience increasingly underestimated speed as speed increased whereas participants with driving experience only underestimated the highest speeds.

As driving speed increased from 5 mph to 65 mph, the estimation of driving speed changed from overestimation to underestimation (speed group effect, F_5,130_ = 66.2, p<0.001). Speed estimations were most accurate in the 25–35 mph range (error: -0.47±0.59). While males and females showed the same pattern across speed groups, the difference between males and females (gender effect) approached significance (F_1,26_ = 3.9, p = 0.058). The interaction between gender and speed group was significant (Fig 4A; F_5, 140_ = 3.1, p = 0.01). Females had higher estimation errors relative to males with the difference greatest at the highest and lowest speeds. There was a significant interaction between driving experience and the speed group as well (Fig 4B; F_5, 140_ = 3.8, p<0.003). Participants without driving experience showed increasing underestimation of speed as driving speed increased, whereas participants with driving experience only underestimated the highest 2 speed groups.

## Discussion

One of the goals of this study was to look at the impact of image scale on the perception of vehicle speed in the real world. Estimation errors were found to be greater with larger image scales. The effect of screen size on speed perception can add to the literature on factors related to simulator fidelity [6]. While it is unclear why underestimation error was larger for the 1:1 image scale than smaller scales, our findings on the trend of error with increasing image size is consistent with the well-known “field size effect” on speed perception–larger apertures make human observers to perceive the speed of moving dot stimuli slower than through smaller apertures [18, 21–23]. It should be noted that those previous vision science studies investigated the speed of random dots on retina, while the subjects in our study estimated the actual vehicle speed in the real world. Because of perceptual constancy mechanisms [24], human visual systems are able to compensate for the difference in retina speed caused by different image scales for the real world images. Specifically, the subjects in our study needed to compensate when viewing the images shown on a 2D display from 25 inches away. It might be possible that observing 0.38 scale images was relatively close to the subjects’ video watching experience with TVs, computer screens, and mobile devices, and therefore their compensation was relatively more accurate. Observing life size images spanning 88 degrees horizontally on a big TV from 25 inches away is not something people would have many experience. Presumably, the combination of lack of experience and the “field size effect” caused larger underestimation of speed for bigger image scales. However, this doesn’t necessarily mean people always underestimate speed when they are physically on the roads. Studies have shown perception of real objects can be different from that for object images shown on display. For instance, perceived size of objects on display is smaller than in the real world [25]. Further studies are needed to investigate whether perceived and verbal reported speed based on displays may be different from verbal reports in the real world.

It is not a surprise that this study found a significant difference in speed estimation between those with and without driving experience. This finding suggests that estimates of speed improve with experience and could be improved with practice. Some experienced participants reported that they relied on the dashed lane markers to estimate vehicle speed so they would not be “tricked” by the slow image motion on both sides in the wide road videos. On the contrary, participants without driving experience were apparently “tricked” by wide road videos. As Fig 3 shows, they had larger underestimation error for wide roads. These findings suggest it may be worthwhile to intentionally add dashed lane markers in simulated scenarios to provide schema for driving speed estimation, if maintaining speed is problematic in simulator studies. Fig 4B shows high speeds have higher estimation error compared to slower speeds in both novice and experienced drivers and this is evident much more drastically in those with no driving experience.

Our study might be able to explain to some extent why the Godley and Tornros’ driving simulator validation studies [11–12] yielded opposite findings on driving speed. The participants in both studies had driving experience. In Godley’s study, the driving speed was always below 37.5 mph (60km/h) and the participants drove slower in the simulator. In Tornros’ study [12] the speed in simulator was above 50 mph (80km/h), and the participants drove faster in simulator. According to our finding (Fig 4B), our subjects with driving experience slightly overestimated speed for low speed range up to 40 mph, which could presumably make one tend to drive slower in a simulator. For speeds in 50 mph group and above, our subjects underestimate the speed, which could presumably make one tend to drive faster in a simulator.

The finding that female participants had larger errors than males (Fig 4A) seems to be consistent with a large body of literature showing that males often outperform females in a variety of spatial orientation tasks including distance estimation [26], which is related to speed estimation. However, it is possible that the gender difference found in this study might not be applicable to driving in the real world. Coluccia & Louse (2004) [26] point out that some gender differences found in simulation studies may not be present in real world studies. Furthermore, gender differences in simulations can change depending on the mental workload the task requires [26].

The findings of this study may have implications for driver training. Those who are well aware of their driving speed are able to set a proper speed for themselves and therefore reduce the chance of an accident. Based on the results, driver training program should pay relatively more attention to speed perception training among the drivers with less driving experience (e.g., drivers who just received their permit). Specific speed perception training programs may also need to be developed or better adapted to target specific driver populations.

The results of this study might have been different (more or less significant) if the sample size were fully balanced in terms of gender and driving experience. As the range of driving experience was wide and the subjects were grouped into two only (with and without experience), the results may be limited regarding the quantitative impacts the level of experience have on speed estimation in simulators. Since perception highly influences behavior, understanding participants’ speed perception based on videos can help interpret their behaviors and performance in simulated driving tasks, or even in real-world driving.

## Supporting information

S1 File Speed estimation data. (XLSX)
